# Supplementary material for: Highly replicated experiments studying complex genotypes using nested DNA barcodes
Source: bioRxiv. 2025 Mar 19:2025.03.18.643964. Preprint. [Version 1] doi: 10.1101/2025.03.18.643964 (PMC11956976; doi:10.1101/2025.03.18.643964)
Supplement: 1 [file NIHPP2025.03.18.643964V1-supplement-1.pdf]

bioRxiv preprint doi: https://doi.org/10.1101/2025.03.18.643964; this version posted March 19, 2025. The copyright holder for this preprint (which was not certified by peer review) is the author/funder. This article is a US Government work. It is not subject to copyright under 17 USC 105 and is also made available for use under a CC0 license.

Supplementary material

Hardcoded barcode	Correct sequence	Error in primer seq.	Incorrect cargo	Primer dimer	SNP/indel
RAD1	**505**	**38**	0	69	0
ERCC4	**450**	**13**	0	20	0
RAD1-neg	**221**	**96**	0	141	0
RAD10	**1405**	**82**	1	163	3
ERCC1	**1494**	**164**	4	37	0
RAD10-neg	**1729**	**43**	0	17	4
RAD14	**709**	**19**	1	33	0
XPA	**1270**	**54**	1	53	2
RAD14-neg	**1985**	**46**	0	22	6

Supplementary table 1. ICR barcodes were characterized as mapping to correct or incorrect gene cargos. Incorrect cargos included those that contained the wrong gene, a primer dimer, or the correct cargo with a SNP or indel. Bold indicates the ICR barcodes that were used in downstream analyses.

Supplementary table 2. Significant differences in normalized barcode abundance for pairs of genotypes. (Included as a separate spreadsheet.)

Supplementary table 3. Strains used in this study. (Included as a separate spreadsheet.)

Supplementary table 4. Plasmids used in this study. (Included as a separate spreadsheet.)

Supplementary table 5. Oligonucleotides used in this study. (Included as a separate spreadsheet.)

bioRxiv preprint doi: https://doi.org/10.1101/2025.03.18.643964; this version posted March 19, 2025. The copyright holder for this preprint (which was not certified by peer review) is the author/funder. This article is a US Government work. It is not subject to copyright under 17 USC 105 and is also made available for use under a CC0 license.

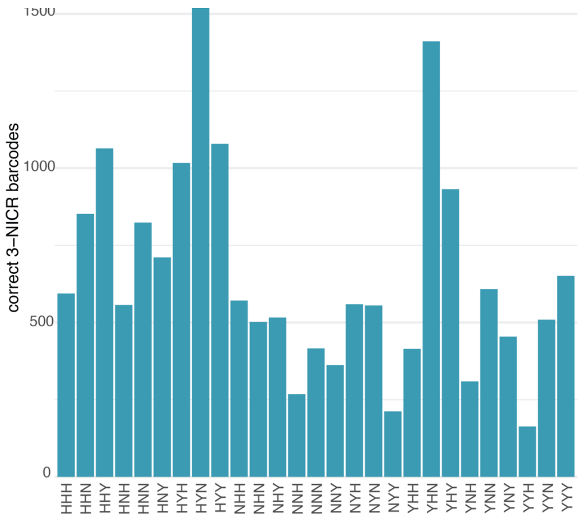

Supplementary figure 1.

Counts of unique 3-NICR barcodes observed in the initial yeast pool. Three-letter genotype codes are as in Fig. 2c.

bioRxiv preprint doi: https://doi.org/10.1101/2025.03.18.643964; this version posted March 19, 2025. The copyright holder for this preprint (which was not certified by peer review) is the author/funder. This article is a US Government work. It is not subject to copyright under 17 USC 105 and is also made available for use under a CC0 license.

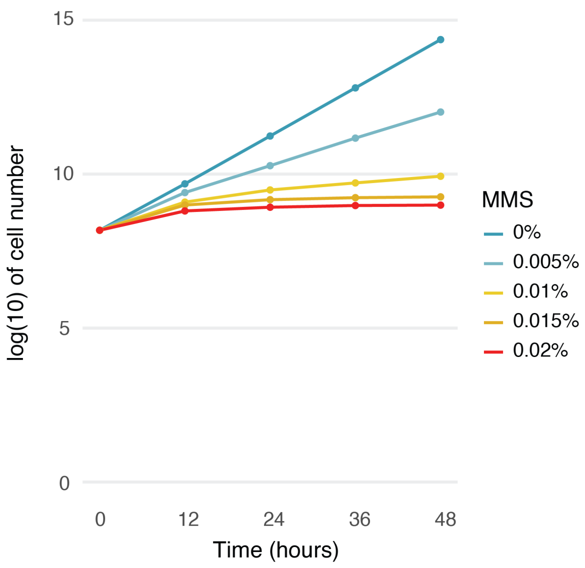

Supplementary figure 2.

Growth in 0%, 0.005%, 0.01%, 0.015%, and 0.02% MMS of the 3-NICR yeast library cultures. Growth was measured every 12 hours as the optical density at 600 nm of each culture, with cultures that reached an optical density of 0.2 or higher diluted to an optical density of 0.025 to prevent them from reaching saturation.
